# Supplementary figures and images for: Comparative analysis of ChIP-exo peak-callers: impact of data quality, read duplication and binding subtypes
Source: BMC Bioinformatics. 2020 Feb 21;21:65. doi: 10.1186/s12859-020-3403-3 (PMC7035708; doi:10.1186/s12859-020-3403-3)

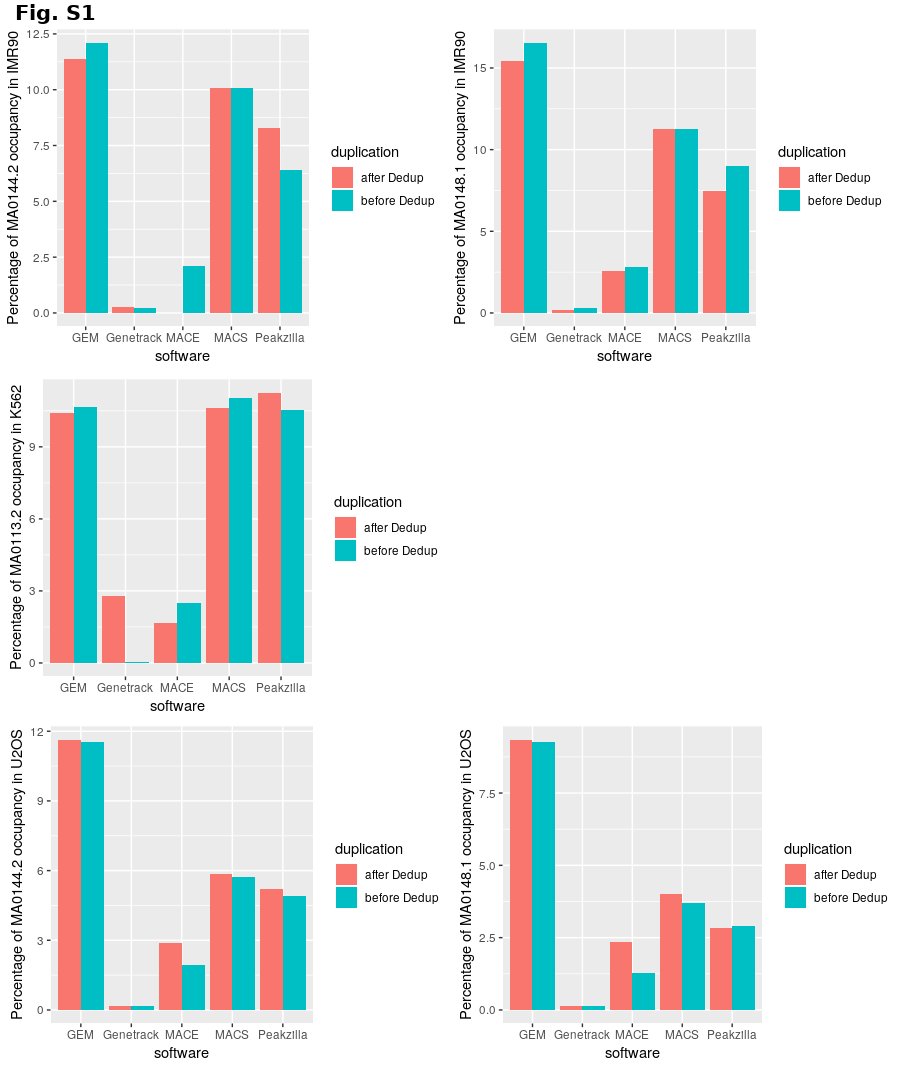

Supplement: Supplementary file 1 — Additional file 1: Figure S1. FOXA1 (JASPAR MA0148.3) and STAT3 (JASPAR MA0144.2) occupancy in IMR90, U2OS, and GBS (JASPAR MA0113.2) in K562 datasets before and after deduplication, as reported by FIMO. Table S1. Total number of peaks/binding events reported by the tools in IMR90, K562 and U2OS datasets. Table S2. Total number of binding events discovered by each peak caller for IMR90, K562, U2OS, after filtering out the PCR duplicates. Table S3. Total GBS motif occupancy (GBS motif hits (p-value <1e-4) reported by FIMO). Table S4. Peak length statistics for GEM, Genetrack, MACE, MACS, and Peakzilla when run on GR ChIP-exo datasets for IMR90, K562 and U2OS cell types. [file 12859_2020_3403_MOESM1_ESM.zip › FigS1.tiff]
